# A Study of the Internal Deformation Fields and the Related Microstructure Evolution during Thermal Fatigue Tests of a Single-Crystal Ni-Base Superalloy

**DOI:** 10.3390/ma17122821

**Published:** 2024-06-10

**Authors:** Cui Zong, Sujie Liu, Guangcai Ma, Yi Guo, Zhaohui Huang

**Affiliations:** 1 National Key Laboratory of Advanced High Temperature Structural Materials, Beijing Institute of Aeronautical Materials, Beijing 100095, China; czong19@hotmail.com (C.Z.); huangzhaohui621@163.com (Z.H.); 2 Institute of Metal Research, Chinese Academy of Sciences, Shenyang 110016, China; sjliu20s@imr.ac.cn (S.L.); gcma@imr.ac.cn (G.M.)

**Keywords:** stress fields, dislocation density distribution, HR-EBSD, thermal fatigue

## Abstract

Ni-base superalloys operate in harsh service conditions where cyclic heating and cooling introduce deformation fields that need to be investigated in detail. We used the high-angular-resolution electron backscatter diffraction method to study the evolution of internal stress fields and dislocation density distributions in carbides, dendrites, and notch tips. The results indicate that the stress concentrations decay exponentially away from the notch, and this pattern of distribution was modified by the growth of cracks and the emission of dislocations from the crack tip. Crack initiation follows crystallographic traces and is weakly correlated with carbides and dendrites. Thermal cycles introduce local plasticity around carbides, the dendrite boundary, and cracks. The dislocations lead to higher local stored energy than the critical value that is often cited to induce recrystallization. No large-scale onset of recrystallization was detected, possibly due to the mild temperature (800 °C); however, numerous recrystallized grains were detected in carbides after 50 and 80 cycles. The results call for a detailed investigation of the microstructure-related, thermally assisted recrystallization phenomenon and may assist in the microstructure control and cooling channel design of turbine blades.

## 1. Introduction

Nickel-base superalloys have been widely used in gas turbines due to their superior mechanical properties at high temperatures. The turbine blades are subjected to cyclic mechanical and thermal loading during operations, and thermal gradients build up in the airfoils and from cooling channels [1]. Due to the fact that temperature and mechanical loadings usually operate simultaneously on turbine blades, many studies have been conducted on the mechanical and thermal–mechanical responses of superalloys [2,3,4], while the effect of temperature alone has attracted less attention. Nevertheless, the study of the effect of cyclic heating and cooling, referred to as thermal fatigue, is an important aspect of secondary orientation studies [5,6,7] and is a useful method for establishing the relationships between the level of deformation and recrystallization [8,9].

Previous studies have revealed some general features of thermal fatigue. Cracks were found to nucleate in hetero-phases (i.e., carbides, oxides, inclusions, etc.) [10,11,12], voids [13], and grain boundaries [14,15] and grow approximately 45° to the dendrite growth direction [16] or along grain boundaries [14]. The crack propagation mode was found to be sensitive to temperature, with a switch from interdendritic propagation to crystallographic propagation along the (111) plane with increasing temperature in a DZ444 nickel base superalloy [17]. The crack growth dynamics were found to be affected by secondary orientations [18,19] with [100] orientations more resistant to crack growth compared with [110] orientations, and the crack growth rates of the two orientations were both accelerated at higher temperatures.

While the above-mentioned studies point to the fact that the stress built up during thermal fatigue plays a critical role in crack nucleation and growth, detailed investigations of the distribution and evolution of the internal deformation fields have scarcely been reported. Indeed, it is difficult to probe the deformation field of thermal fatigue tests using conventional routines due to the specific experimental setup and the fact that deformations arise internally rather than being imposed externally. The cross-correlation-based HR-EBSD method [20,21] may provide a solution to such problems. This method exploits the fact that lattice distortions induce shifts in diffraction patterns and calculates elastic strain, stress, and dislocation density distributions based on the measured pattern shifts. The utility and capacity of HR-EBSD have been demonstrated by its wide adoption in the study of martensite transformation [22], slip transfer at the grain boundary [23], thermal mechanical strains near carbides in superalloys [24,25], and in situ micro-pillar compression [26], to name a few. In this study, we use HR-EBSD to study the distribution of internal stress and dislocation density distributions that develop in the notch tip, carbides, and dendrites after thermal fatigue tests. The information is then used to investigate the slip activity that correlates crack development and the evolution of dislocation densities discussed in the context of the detected recrystallization phenomena.

## 2. Materials and Methods

The sample used in this research was a model alloy supplied by the Beijing Institute of Aeronautical Materials, and the chemical composition of the material is shown in Table 1. The directionally solidified single-crystal ingot was solution heat-treated in a vacuum at 1300 °C for 2 h followed by three periods of 4 h of aging at 1120 °C, 1080 °C, and 900 °C sequentially. All cooling was air cooling, and temperature was controlled to be within ±10 °C.

**Figure 1:**
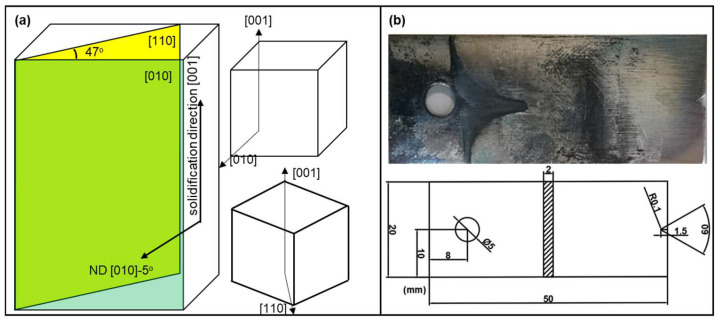
(**a**) Schematics of the sampling location on the cast ingot to obtain the [100] and [110] secondary orientation samples; the upper cube indicates that the sample was prepared such that the flat face is along the [010] direction and the bottom cube indicates that the sample’s flat face is along the [110] direction. (**b**) Dimension and geometry of the thermal fatigue sample.

To characterize the crystal orientation of the ingot, a small piece of sample was cut from the ingot and inspected using EBSD (FEI Apreo, Thermo Fisher Scientific, Waltham, MA, USA). The result indicated that the solidification direction was along the crystallographic [001] direction, and the ingot normal direction, as schematically shown in Figure 1a, deviated from the crystal [010] direction by 5°. With this information, the [110] secondary-oriented sample can be obtained by sampling along the plane at 50° to the ND plane, but due to alignment error, the actual cutting angle was 47°, resulting in a 3° misalignment to the [110] crystallographic direction. However, we assume that such a small angular deviation is insignificant and henceforth refer to the secondary orientations as [010] and [110] orientations.

The thermal fatigue sample was prepared using wire cutting and the notch was refined by a CNC grinding machine to ensure improved smoothness of the notch surface with lower residual stress. The design of the samples is shown in Figure 1b. The thermal fatigue tests were performed using an LRP1200 hot and cold fatigue system (Jinan test machine Co. Ltd., Jinan, China) with one thermal cycle defined as heating up to 800 °C and holding for 8 min followed by water quenching to 20 °C in 30 s. EBSD investigations were performed after 20, 50, and 80 cycles to study the local deformation fields associated with the thermal strain and microstructure evolutions. The EBSD experiments were conducted at 20 kV and 14 nA under a 15 nm working distance with a step size of 0.5 µm. The resultant EBSD maps were analyzed without map cleaning. At 800 °C, oxide scales built up during the thermal fatigue test, and to ensure sufficiently high quality for the EBSD study, the samples were ground using 5000 grit sandpaper followed by diamond and colloidal silica polishing, as well as final electro-polishing at 15 kV, where the samples were soaked in a 10% perchloric acid + 90% ethanol solution for 50 s at −20 °C. The microstructures were also inspected using a Zeiss Axio Observer Z1 optical microscope (Carl Zeiss AG, Baden-Württemberg, Germany).

To study the evolution of the thermally induced internal deformation fields that accompany crack initiation and growth, we used the cross-correlation-based EBSD method, i.e., HR-EBSD, which calculates elastic strain fields based on the relative shifts of the electron back-scatter diffraction patterns. In brief, the displacement gradient tensor was obtained from the measured shift in diffraction patterns. The symmetric part of the displacement gradient tensor, representing strain, was integrated with the stiffness matrix to obtain stress. This analysis was performed using CrossCourt4 software (Ver. 4.0) and the underlying theory can be found in reference [21]. A second pass of cross-correlation was performed to obtain strain fields with improved precision [20]. These analyses were performed after 20, 50, and 80 thermal cycles for each of the secondary orientation types.

## 3. Results

**Figure 2:**
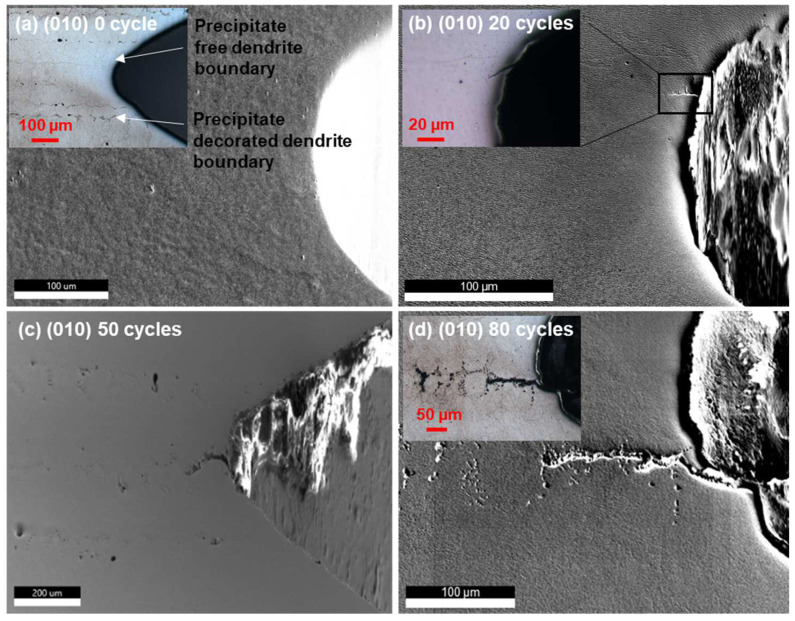
SEM images of the (100) secondary orientation sample revealing the microstructure evolution around the notches subjected to various cycles of thermal fatigue. The insets are optical images showing different perspectives of the microstructures and cracks near the notch. Note: “precipitate free” in this paper refers to “precipitate is not visually identifiable by the characterization tools used”.

**Figure 3:**
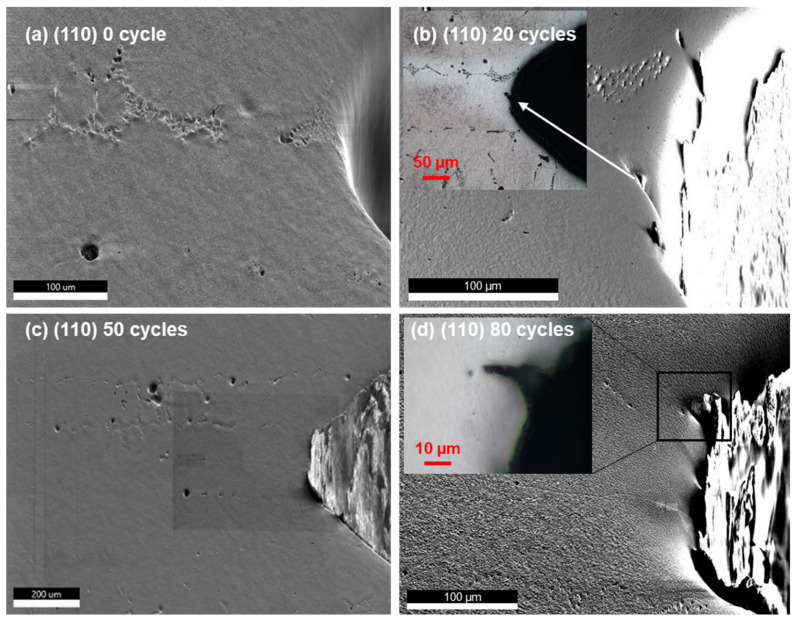
SEM images of the (110) secondary orientation sample revealing the microstructure evolution around the notches subjected to various cycles of thermal fatigue. The insets are optical images showing different perspectives of the microstructures and cracks near the notch.

The SEM micrographs of the notch tip region of the [010] secondary orientation samples thermally fatigued for 0, 20, 50, and 80 cycles are shown in Figure 2, overlaid with some optical images showing the dendrite and precipitate (TaC) distributions more clearly. Note that Figure 2a,b shows results obtained from the same sample, whereas Figure 2c,d shows a different sample prepared from the same ingot with the same secondary orientation. The ~20 µm long crack after 20 thermal cycles (Figure 2b) seems to originate from the notch tip below a precipitate-free dendrite boundary and runs diagonally, whereas the cracks in Figure 2c,d seem to originate by the precipitate string (i.e., a string of precipitate) intersecting the notch tip and runs horizontally following the precipitate string. In contrast, for the (110) secondary orientation samples (Figure 3), cracks seem to initiate from the tip of the notch independent from either the dendrite arms or the precipitate strings and the crack length does not seem to develop significantly; as thermal fatigue cycles build up to 80 cycles, a different crack growth behavior is observed compared to the (010) samples.

**Figure 4:**
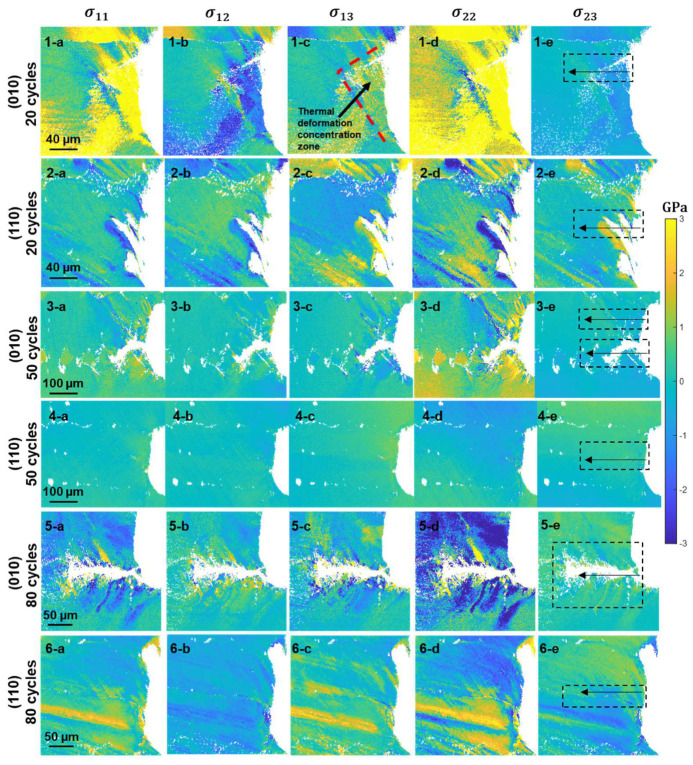
HR-EBSD stress fields around the notches measured on the surface of the samples. Each row, as has been labeled, is a specific secondary orientation sample subjected to a specific thermal cycle, and each column is a component of the stress tensor. The dashed boxes indicate the location and size of a zone scan to obtain the stress profiles shown in Figure 5. Each row corresponds to the indicated type of secondary orientation subjected to the indicated number of thermal cycles and each column corresponds to a type of indicated stress tensor components.

The stress fields obtained by HR-EBSD are tabulated in Figure 4. The magnitude of the local stress concentrations in some stress tensor components, e.g., Figure 4(1-d), seems to be much higher than the macroscopic yield stress (~850 MPa). This is not impossible as long as mechanisms exist that pin the dislocations in place, in which case the local stress can build up to any level below the theoretical strength. This principle is similar to the effect of testing a dislocation-free crystal to obtain the theoretical strength [27]. Nevertheless, instead of interpreting the results based on the absolute magnitude, we analyzed the results based on the pattern of distributions, and comparisons were made in terms of the relative magnitude.

**Figure 5:**
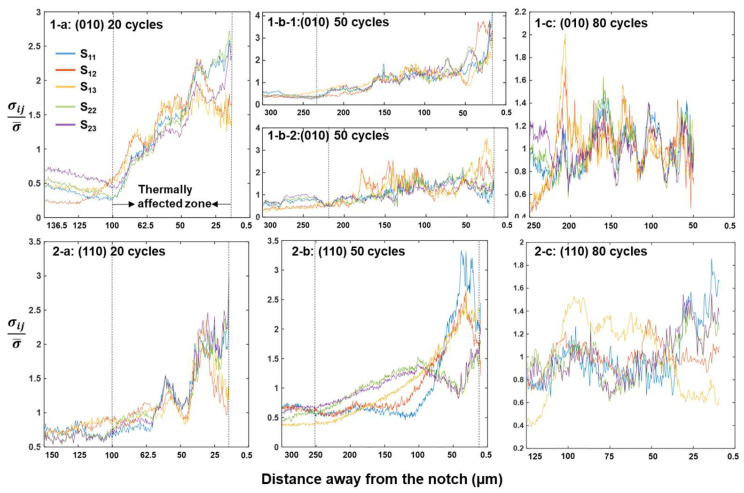
Thermal stress distribution ahead of the notches. (**1-a**–**1-c**) are those for (010) orientations and (**2-a**–**2c**) are those for (110) orientations. The zone scan locations and the scanning directions (away from notch) are indicated in Figure 4.

It is apparent from the stress maps in Figure 4 that the thermal stresses are distributed asymmetrically across both the dendrite arm (1-a–1-e, 6-a–6-e) and the precipitate string (2-a–2-e). Such an asymmetrical stress field may help in promoting crack nucleation, as the stress is not compatible across the boundary; however, as can be seen from Figure 2b and Figure 3b, the cracks seem to initiate independent of such microstructure features. On the other hand, 3-a–3-e and 5-a–5-e demonstrate crack propagation along precipitate strings. Under such circumstances, the stress fields seem to distribute relatively evenly across the cracked precipitate strings. It is interesting to note that in such cases where the crack propagates along the precipitate string, the normal terms of the stress tensor are distributed antisymmetrically and the shear terms are distributed symmetrically across the crack surface. It can be seen from Figure 4(5-a–5-e) that local stress spikes radiate from the crack surface, indicating localized shear that promotes dislocation emissions from the crack tip as the crack develops over the thermal cycles. Another feature that we noticed from Figure 4 is that the (010) 20-cycle sample (1-a–1-e) demonstrated more significant internal thermal stress compared to other maps. Further inspection of the stress maps indicates that there seem to be two intersecting slip bands, and the boundaries of those slip bands led to discontinuity of the thermal strain field, which confined the thermal strain into a thermal deformation concentration zone to the right-hand side of the intersections (indicated in Figure 4(1-c)). This feature may give rise to the excessive thermal stress that built up, as seen in Figure 4(1-a–1-e), and helped promote crack nucleation along the upper slip band away from the dendrite arm. Unfortunately, as the samples were repolished after each set of thermal cycles (to remove the oxidants), slip traces were not directly observable on the sample surface. In the following, as the internal thermal stress has rarely been measured experimentally at this level of resolution, we would like to first quantitatively demonstrate the profile of the thermal stress field ahead of the notch tip in Figure 5. Then we will show how we conducted the slip trace analysis with the aid of the stress maps to understand the slip activity.

**Figure 6:**
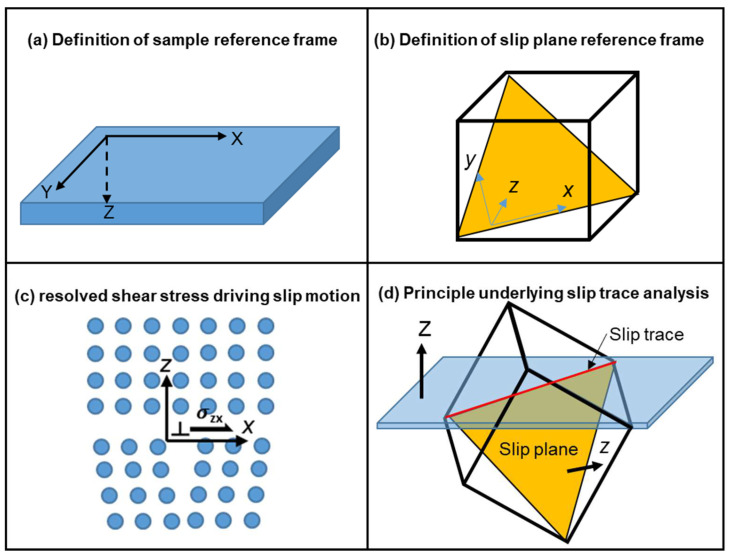
Schematics demonstrating the principles of stress field rotation and slip trace analysis.

The stress profiles shown in Figure 5 were obtained by a zone scan where the zone size and location, together with the scanning direction, are indicated in Figure 4. These curves quantitatively indicate the distribution of the residual thermal stress and therefore provide a lower bound estimate (stress may be relieved after cooling) of the thermally affected zone size. Each curve in Figure 5 represents a column-averaged absolute stress tensor component of the zone and is normalized by the absolute mean of all data in the zone. The stress maps of the (010) 50-cycle sample were divided into two parts, with 1-b-1 representing the stress profiles away from the carbide string and 1-b-2 representing those centering the carbide string. It can be seen for both types of orientations that the residual thermal stress decays with distance away from the notch. After 20 thermal cycles, the thermally affected zone spans a ~100 µm range and stretches to ~200 µm after 50 cycles. 1-b-1 and 1-b-2 show similar stress distributions overall, indicating that the presence of the carbide string does not seem to modify the thermal stress field significantly apart from some local stress spikes that arose due to stress concentrations at the carbide interface. In contrast to the exponential decay stress profiles shown in 1-a–1-b and 2-a–2-b, those shown in 1-c and 2-c mainly feature stress spikes oscillating around the mean stress. Upon interpreting such stress profiles together with the stress field images in Figure 4, it emerges that such oscillating stress patterns are due to local dislocation emissions from either the crack surface (1-c) or the dendrite arm (1-d). It is likely that after 80 thermal fatigue cycles, the thermal stress has been redistributed from the notch to local slip bands. We now proceed with slip trace analysis, as shown in Figure 6, to study the meso-scale dislocation activities during thermal fatigue.

**Figure 7:**
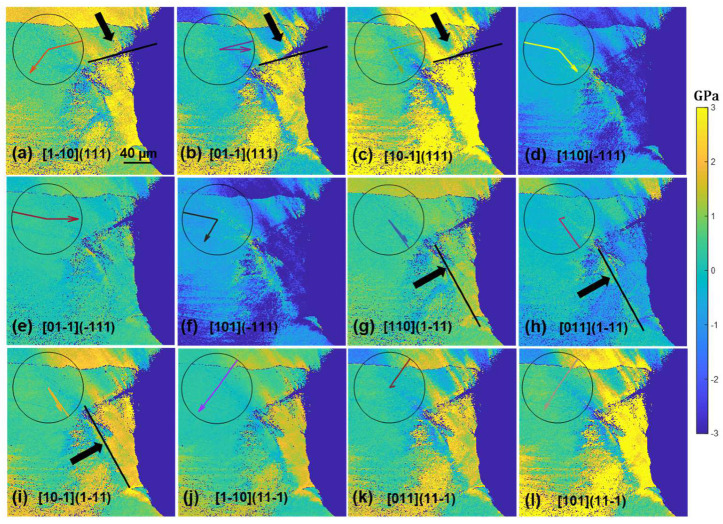
Resolved shear stress maps of the 12 slip systems of the (010) secondary-oriented sample subjected to 20 thermal cycles. The overlaid clock chart demonstrates the slip trace direction (solid line) and Burger’s vector direction (solid arrow) plotted in the sample reference frame. By looking at the solid lines on the clock chart, one can infer the meso-scale slip trace if the corresponding dislocation pile-ups were formed. The scale bar shown in the first image applies to all other images.

**Figure 8:**
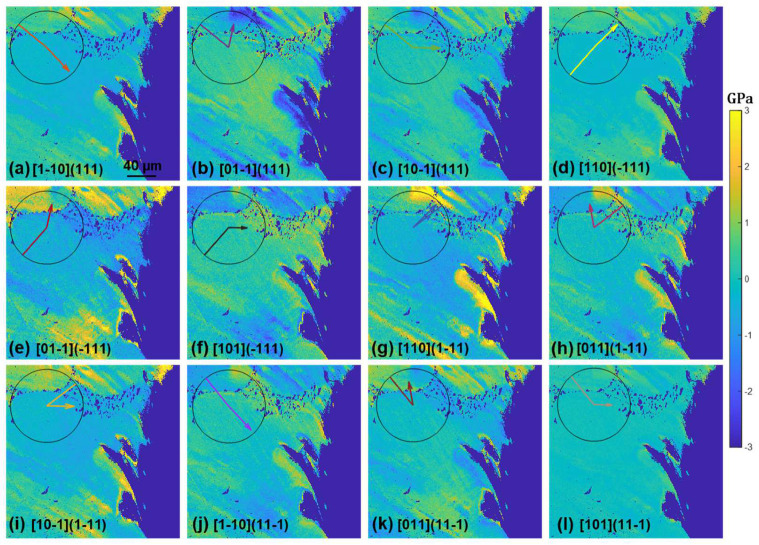
Resolved shear stress maps of the 12 slip systems of the (110) secondary orientation sample subjected to 20 thermal cycles. The overlaid clock chart demonstrates the slip trace direction (solid line) and Burger’s vector direction (solid arrow) plotted in the sample reference frame. By looking at the solid lines on the clock chart, one can infer the meso-scale slip trace if the corresponding dislocation pile-ups were formed. The scale bar shown in the first image applies to all other images.

**Figure A1:**
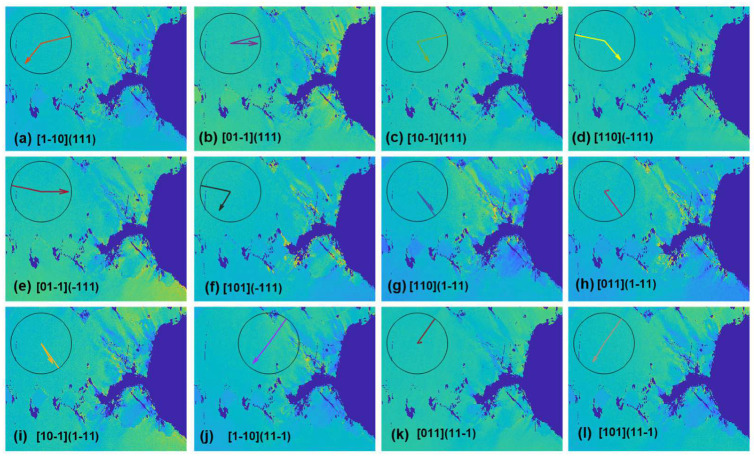
Resolved shear stress maps of the (010) secondary-oriented sample subjected to 50 thermal cycles. The overlayed clock chart demonstrates the slip trace direction (solid line) and Burger’s vector direction (solid arrow) plotted in the sample reference frame.

**Figure A2:**
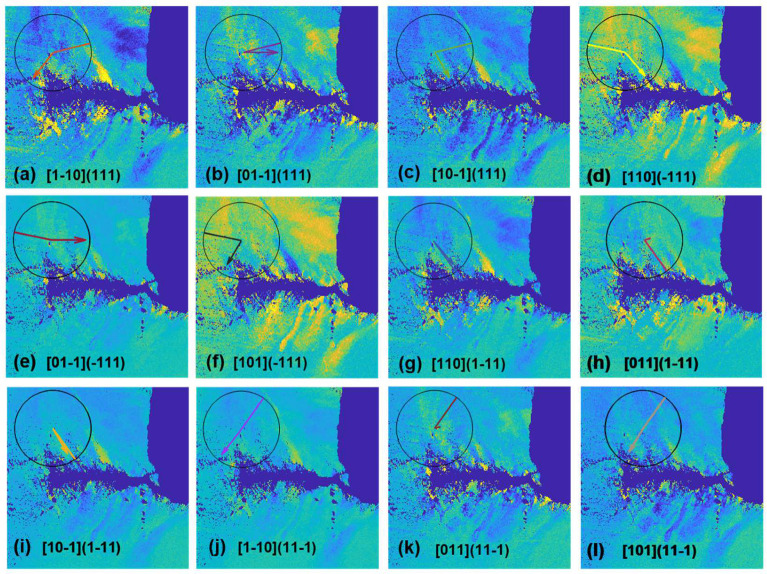
Resolved shear stress maps of the (010) secondary-oriented sample subjected to 80 thermal cycles. The overlayed clock chart demonstrates the slip trace direction (solid line) and Burger’s vector direction (solid arrow) plotted in the sample reference frame.

**Figure A3:**
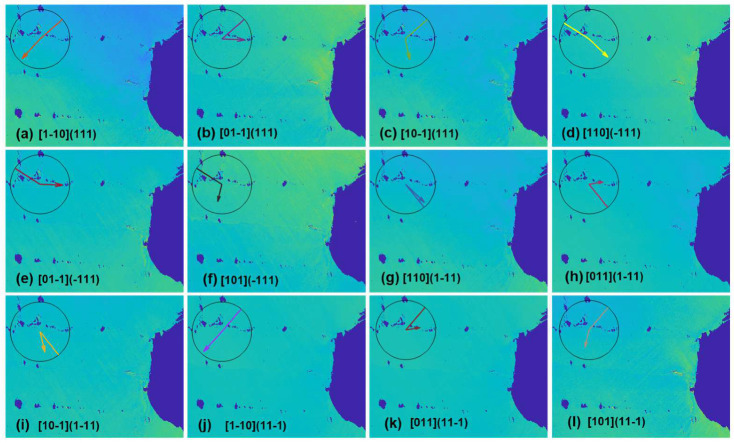
Resolved shear stress maps of the (110) secondary-oriented sample subjected to 50 thermal cycles. The overlayed clock chart demonstrates the slip trace direction (solid line) and Burger’s vector direction (solid arrow) plotted in the sample reference frame.

**Figure A4:**
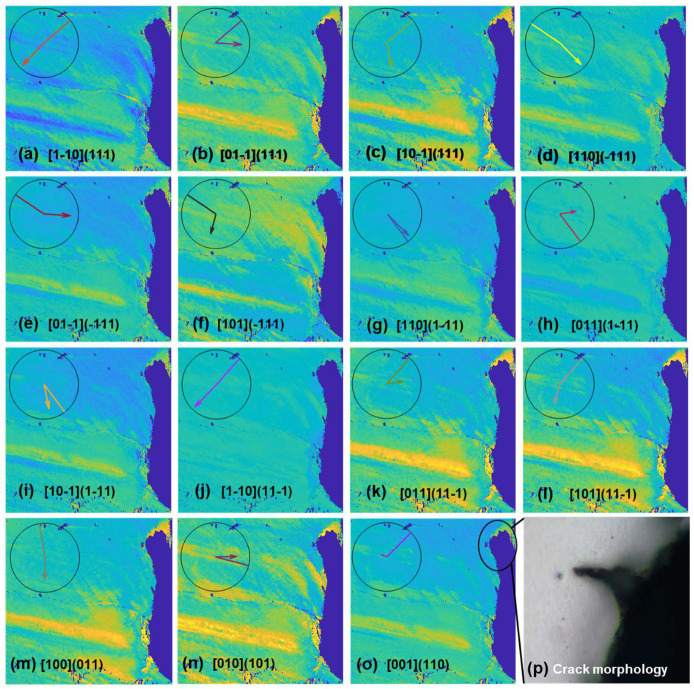
Resolved shear stress maps of the (110) secondary-oriented sample subjected to 80 thermal cycles. The overlayed clock chart demonstrates the slip trace direction (solid line) and Burger’s vector direction (solid arrow) plotted in the sample reference frame. The location of the crack on the stress maps, as indicated by the circle on (**o**), has low diffraction pattern quality due to severe deformation and thus was filtered out, and the correct shape of the crack is shown in (**p**).

Figure 6 schematically indicates the general principles of slip trace analysis and stress field rotations to resolve shear stress on slip systems. The sample reference frame, where the stress maps in Figure 4 were generated, is shown in Figure 6a. To obtain resolved shear stress on slip systems, we define a second set of slip systems where the x-axis is along Burger’s vector direction and the z-axis is perpendicular to the slip plane, as shown in Figure 6b. Rotation of the stress field from the sample reference frame to the slip reference frame is performed using the following operation:

(1) $$\sigma_{slip}=R^{-1}\sigma_{sample}R$$

(2) $$R=\left(\begin{array}{ccc} b_{1} & l_{1} & n_{1}\\ b_{2} & l_{2} & n_{2}\\ b_{3} & l_{3} & n_{3} \end{array}\right)$$

where $\hat{b_{i}}$ and $\hat{n_{i}}$ are the units for Burger’s vector and slip plane normal directions expressed in the sample reference frame, and $\hat{l_{i}}=\hat{n_{i}}\times \hat{b_{i}}$. Once the stress tensor is rotated from the sample reference frame to the slip reference frame, the $\sigma_{zx}$ tensor component represents the resolved shear stress of the slip system (Figure 6c). For the nickel superalloy, there are 12 such rotations, and the resultant shear stress maps are shown in Figure 7 and Figure 8 to demonstrate the results for (010) and (110) orientations after 20 thermal cycles. To avoid showing images of the same type repetitively, the shear stress maps after 50 and 80 cycles are shown in Appendix A Figure A1, Figure A2, Figure A3 and Figure A4. As has been mentioned above, slip traces were removed due to mechanical and chemical polishing after each set of thermal cycles. To infer the slip trances that were activated, we explore the concept that slip traces arise due to the intersection between the slip plane and the sample surface (Figure 6d) and conducted cross products between the slip plane normal and the sample normal to obtain the slip trace, which is plotted together with Burger’s vector directions in the sample reference frame to obtain the overlaid “clock chart” shown in Figure 7 and Figure 8. One can compare the solid line on the clock chart, which represents the hypothetical slip trace, with the crack trace and possibly the lines of stress concentrations to infer the crystallographic information of those features. We see from Figure 7 that after 20 cycles, the crack located at the top right corner below the dendrite arm seems to follow a (111) plane trace; however, due to symmetry, there are three possible slip systems that converge with the crack trace (indicated by the arrows in Figure 7a–c). We also notice that the resolved shear stress field seems to terminate at a certain boundary indicated by the arrows in Figure 7g,h, and this hypothetical boundary also seems to converge with the orientation of the shear stress bands nearly perpendicular to the crack surface. As indicated by the clock chart, this boundary likely arises from one of the (1-11) slip systems and intersects with one of the (111) slip systems. As Figure 7 shows the shear stress resolved on slip systems, each image represents the local resolved shear stress that drives the corresponding dislocation motion. Without dedicated micro-scale experimental investigations, the specific slip systems can be identified by the highest local resolved shear stresses, and in this case, it looks like the [10-1](111) and [10-1](1-11) slip systems were activated by thermal stress, forming a local thermally affected zone. The excessive thermal stress built up in the zone led to cracking along the (111) plane. Further inspections of the shear stresses indicate that the cracking of the (111) plane may be assisted by the shear and activation of the (1-11) slip systems. This is indicated by the shear stress bands nearly perpendicular to the crack surface where the shearing of the (1-11) plane exerts dilatational stress on the (111) slip plane. Similar dislocation emissions may be inferred from the samples tested for 50 and 80 cycles (Appendix A Figure A1 and Figure A2) where the crack propagates along the precipitate string and is accompanied by emissions of dislocations on the (1-11) plane.

Applying the same analysis to Figure 8, we see that the crack likely formed on the [01-1](111) slip system judging from the slip trace analysis and the relative magnitude of the shear stress. It is interesting to note that although extensive shear stress bands of either (111) or (11-1) types had possibly emitted from the precipitate string, no crack was observed to initiate from these precipitates. The (1-11) type dislocations can also be seen to initiate from along the dendrite arms in Appendix A Figure A4, and similar to the case shown in Figure 8, crack nucleation seems to be independent of the dendrite arm. The crack in this case seems to follow a cubic slip system as the slip trace analysis reveals in Appendix A.

**Figure 9:**
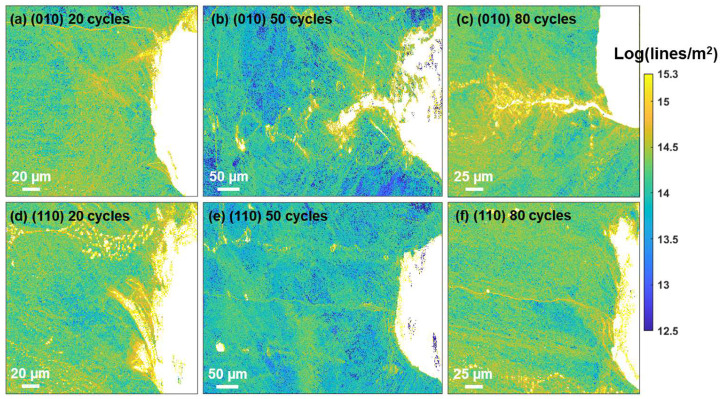
GND density distributions inspected after 20, 50, and 80 thermal fatigue cycles for (010) (**a**–**c**) and (110) (**d**–**f**) secondary orientations.

The above-mentioned elastic field analysis is helpful in identifying the crystallographic nature of the slip traces and cracks and has been applied extensively to deformation twinning analysis [28,29,30,31]. To understand the accumulation of plasticity over the course of thermal fatigue, geometrically necessary dislocation (GND) maps are shown in Figure 9. The dislocation maps were calculated by solving Nye’s dislocation tensor [32] using the measured rotation gradients. Detailed methods using rotations measured in 2D and 3D can be found in references [33,34].

As repeated heating–cooling cycles were applied to the samples, the thermal stress resulted in dislocation emissions at cracks, dendrites, and precipitates, as can be seen in Figure 9. On the other hand, the elevated temperatures also facilitate dislocation annihilation [35], so Figure 9 represents a residual dislocation distribution, which agrees reasonably well with the stress maps shown in Figure 4. Comparatively more significant dislocation activities seem to be associated with the cracks, as is most evident from b and c, and these dislocations form bands and cells, indicating the crack propagates progressively and in a ductile manner. The elevated GNDs (>3 × 10^14^) were found in a range between ~6 and 60 µm across the crack surface as measured in b and c. GNDs were found to form a ~1–6 µm shell encapsulating the precipitates and a few microns across the dendrite arms.

## 4. Discussion

The microstructures of the tested material consist of dendrites and strings of TaC precipitates intersecting the notches. Those microstructure features are prone to thermal deformation concentrations as has been revealed by the EBSD stress and dislocation density maps. However, cracks are weakly correlated with those features and have been observed to form independent of dendritic arms (Figure 7) and precipitates (Figure 8). The nucleation of thermal fatigue cracks was often cited to be oxidation-assisted [14,17,36], where the oxygen concentration assisted by the elevated stress field at the notch tip leads to crack initiation. While it is not clear how the dendrites and precipitates are less responsive to the oxygen-induced cracking mechanism, it does seem clear that crystallographic fractures, i.e., crack initiation along slip planes, set in relatively easier than fractures at dendrites and precipitates. 

The crystallographic feature of the cracks can be identified by the combination of slip trace and locally resolved shear stress analysis. This method has been applied to the analysis of void growth [37] and is especially useful for thermal fatigue analysis where the external loading is absent and traditional Schmid factor analysis is not applicable. The stress field demonstrated above needs to be interpreted with care. These stresses are the residual stresses measured after thermal fatigue testing. It has been shown that the stress fields can be quite different during mechanical holding and after unloading [37,38], involving stress relief and/or local reversal of the directions of stresses. Nevertheless, the stress profiles shown in Figure 5 provide an idea of the size of the thermally affected zone, which may be useful for assisting engineering design. Both the (010) and (110) samples demonstrate an exponential decay of stresses away from the notch, as evidenced by Figure 5a,b, and the stress tensor components vary between the two orientations. These variations arise from anisotropy of the elastic constants to which thermal stress is directionally proportional [18]. These variations lead to different levels of local resolved shear stress on slip systems (Figure 7 and Figure 8), which in turn give rise to the excitation of different types of dislocations, contributing to the secondary orientation effect. Earlier research reported that for the (010) orientation, cracks grow slower than that for the (110) orientation [18], whereas in the current research, the trend seems to be reversed, as for the (110) orientation, a crack of ~20 µm was observed after 80 cycles (Figure 3) compared to the crack of ~130 µm for the (010) orientation (Figure 2). Note that the current tests were conducted at a lower temperature and that temperature is known to affect the fracture mode [17]. On the other hand, the fact that the longer crack of the (010) orientation follows the precipitate string (Figure 2d) and the shorter crack of the (110) orientation is independent of precipitates renders the effect of the secondary orientation less conclusive in the current study.

**Figure A5:**
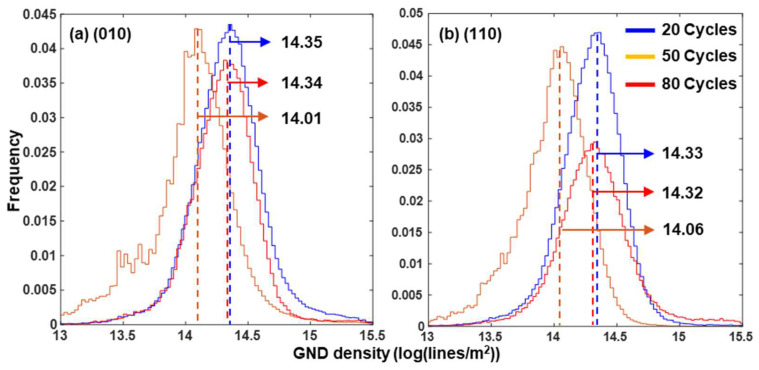
Histograms of the GND density distributions of the (010) and (110) orientations after 20, 50, and 80 thermal cycles. The modes of the histograms are indicated by the arrows and numbers.

It is important to note that elevated dislocation densities have been observed at dendrite arms, precipitate strings, and cracks. The peak of the GND density histogram (Appendix A Figure A5) shifted to ~2.2 × 10^14^ at 80 thermal cycles from ~1.3 × 10^14^ at 50 cycles for both orientations (note that we avoid drawing a comparison with the 20-cycle samples as the tests were conducted on different samples). This trend suggests that the dislocation built up due to thermal stresses was faster than the thermally assisted dislocation annihilations at 800 °C. These high local dislocations that built up raise the question regarding the possibility of recrystallization that is detrimental to single-crystal gas turbines operating at elevated temperatures. The stored energy (in the form of dislocations and dislocation entanglements) in these samples was estimated following the work of Kocks et al. [8,39,40]:

(3) $$E_{st}=\alpha \mu b^{2}\rho \ln\frac{R_{e}}{b}$$

where α equals 1/4π for screw dislocation and 1/4π(1−v) for edge dislocations with a mean value of 0.1 was adopted for dislocations of a mixed type [40]. b is Burger’s vector and *ρ* is the dislocation density. $R_{e}$ is the mean average of the dislocation strain field range and is most commonly approximated as the mean dislocation spacing, 1/ρ [40]. µ is the shear modulus and is set to 96.59 GPa according to a sample supplier.

**Figure 10:**
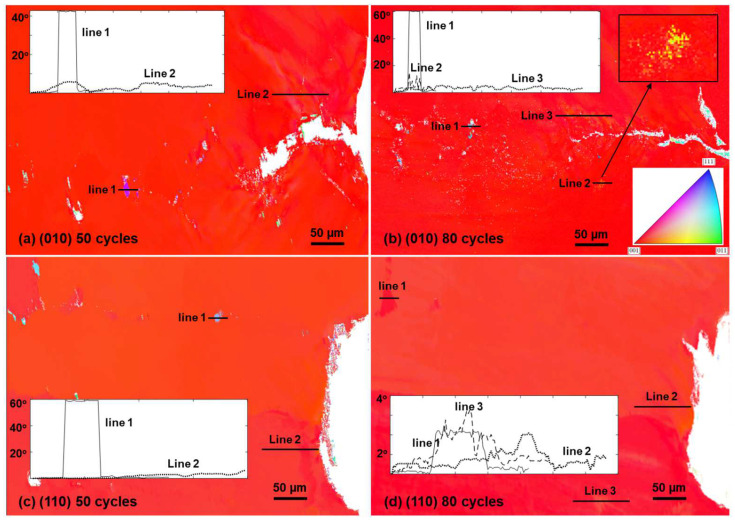
IPF-Y maps showing the orientation gradients after thermal cycles. Locally high orientation gradients were probed using line scan and misorientations were calculated along the line relative to the start point. Results of the line scans are shown in the insets. (**a**,**c**) Tested samples with local recrystallized grains; (**b**) the tested sample with local high misorientation zones but without defined grain boundaries; (**d**) the tested sample without recrystallization or local high misorientation zones.

**Figure A6:**
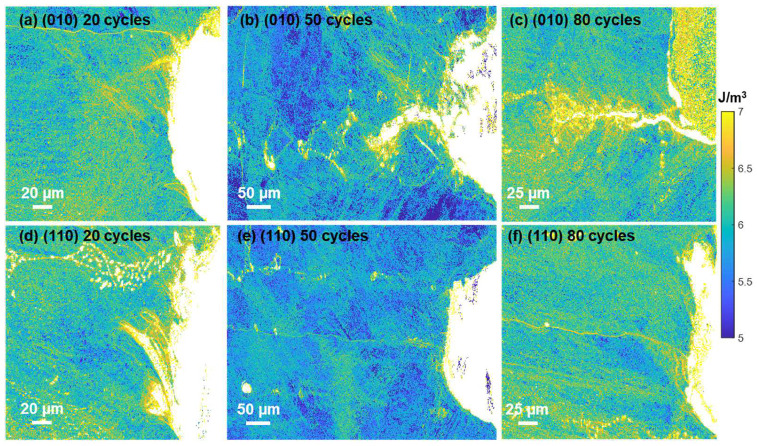
Stored energy distributions of (010)- and (110)-orientated samples after 20, 50, and 80 thermal cycles.

By applying Equation (3) to the dislocation maps shown in Figure 9, stored energy distribution maps were obtained and are shown in Appendix A Figure A6. The stored energy distribution follows the pattern of the dislocation density distribution, and high stored energy concentrations are localized to the cracks, precipitates, and dendrite arms, indicating a strong tendency of recrystallization at these defects. The magnitude of the distributions ranges from 10^6^–10^7^ J/m^3^ and falls within the critical stored energy range [41] for Inconel 718 and higher than that of a Ni-13Co-16Cr type powder metallurgy superalloy [8] and a PWA1483 single-crystal superalloy [9]. Large-scale recrystallization was not found at the elevated stored energy concentrations, possibly due to the mild temperature, but traces of recrystallization were indeed observed after 50 and 80 thermal cycles and the results are shown in Figure 10. There seem to be two types of recrystallizations. The first is those that formed along the precipitate strings and possibly formed in the area in between the carbides. These are indicated by line 1s in Figure 10a–c, featuring recrystallized grains that are 40° or 60° misorientated compared to the surrounding matrix (note that the recrystallized grains in Figure 10c were absent in Figure 10d, and these were possibly polished away). This type of recrystallization seems more populated as multiple such features were identified at both 50 and 80 thermal cycles. The second type of recrystallization was identified at the location indicated by Line 2 in Figure 10b. This kind of recrystallization features a local zone of higher magnitude of misorientations approaching 10° but without a clear shape of a sub-grain boundary (shown in the inset of Figure 10b).

The second type of recrystallization (or maybe it is more appropriate to name such features the local zone of high misorientation) was found at a distance away from the crack instead of at the immediate neighborhood of the cracks where the stored energy and stresses are both higher. As indicated by line scan 2 in Figure 10a and line scan 3 in Figure 10b, the local misorientations near the cracks are around ~10°. It may be the case that recrystallization requires a proper combination of local strain and stored energy [8] and/or the formation of locally recovered subgrains [42]. The current results challenge a previous conclusion that recrystallization in this type of material sets in at temperatures above 1000 °C [43] and points to the fact that recrystallization requires a suitable combination of temperature and dislocation density (stored energy), which is possibly additionally affected by the level of local strains. The relationship between the recrystallization temperature and the critical dislocation density level has been established in Cu and Ag [40,44], and it is necessary to investigate such relationships in future work in the current material system such that the design and service criteria can be properly revised and a better casting strategy can be designed.

Finally, we note that in this research, we employed the HR-EBSD technique to study the local stress field evolutions due to thermal fatigue. The distributions of the stress fields were interpreted with the aid of a meso-scale slip trace analysis. While the deformation compatibility across γ-γ′ phases plays an important role in the deformation of the Ni-base superalloy, such an investigation requires a compatible technique (e.g., TEM) and is beyond the scope of this study.

## 5. Conclusions

The internal deformation fields of a single-crystal nickel-base superalloy that developed during thermal fatigue tests were investigated using HR-EBSD, and based on the experimental observations and data analysis, the following conclusions can be drawn:

(1) Higher levels of micro residual stress arise as a consequence of dislocation generation and interactions with microstructures. Thermal fatigue at 800 °C leads to local stress concentrations that decay exponentially away from the notch. The thermally affected zone spans over a ~100 µm range after 20 cycles and expands to ~200 µm after 50 cycles. Such exponential decay diminishes after 80 cycles due to longer crack length.

(2) The variations in the local stress tensor lead to different levels of resolved shear stresses on slip systems and therefore distinct fracturing behavior of secondary orientations, as have been revealed by slip trace and local resolved shear stress analysis.

(3) The initial cracking follows the crystallographic plane and may not be affected by dendrites and precipitates. The cracks that developed along the carbide string in the (010) sample grew significantly longer than the crack developed in the (110) sample that did not follow carbides or dendrites.

(4) Thermal fatigue leads to elevated GNDs at cracks, carbides, and dendrites. The mode of the GND density histogram shifted from ~1.3 × 10^14^ (lines/m^2^) after 50 thermal cycles to ~2.2 × 10^14^ (lines/m^2^) after 80 cycles. Such local elevated dislocation density provides the precursors and driving force for recrystallization.

(5) Although the thermal fatigue temperature (800 °C) is relatively mild, the development of internal dislocation density can be significant and provide stored energy well above the critical values for recrystallization.

## Figures and Tables

**Table 1:** Chemical composition of the single-crystal model alloy.

	C	Cr	Co	W	Al	Ta	Mo	Ti	Hf	B	Re	Ni
wt %	0.05	7.05	7.5	5.4	6.24	6.58	1.51	1.5	0.16	0.0047	2.96	Bal

## Data Availability

The raw/processed data required to reproduce these findings are available upon request.
